# Cancer Risks in Vitiligo Patients: A Nationwide Population-Based Study in Taiwan

**DOI:** 10.3390/ijerph15091847

**Published:** 2018-08-27

**Authors:** Cheng-Yuan Li, Ying-Xiu Dai, Yi-Ju Chen, Szu-Ying Chu, Tzeng-Ji Chen, Chen-Yi Wu, Chih-Chiang Chen, Ding-Dar Lee, Yun-Ting Chang

**Affiliations:** 1 Department of Dermatology, Taipei Veterans General Hospital, Taipei 11217, Taiwan; Proteinm@yahoo.com.tw (C.-Y.L.); daiinxiu@gmail.com (Y.-X.D.); sychu2@vghtpe.gov.tw (S.-Y.C.); chenyiok@gmail.com (C.-Y.W.); docs1.tw@yahoo.com.tw (C.-C.C.); ddlee@vghtpe.gov.tw (D.-D.L.); 2 School of Medicine, National Yang-Ming University, Taipei 11221, Taiwan; yjchenmd@vghtc.gov.tw (Y.-J.C.); tjchen@vghtpe.gov.tw (T.-J.C.); 3 Department of Dermatology, Taichung Veterans General Hospital, Taichung 40705, Taiwan; 4 Department of Family Medicine, Taipei Veterans General Hospital, Taipei 11217, Taiwan; 5 Institute of Public Health, National Yang-Ming University, Taipei 11221, Taiwan

**Keywords:** bladder cancer, breast cancer, malignancy, prostate cancer, Taiwan, thyroid cancer, vitiligo

## Abstract

Vitiligo is an autoimmune disease characterized by destruction of melanocytes and associated with other autoimmune disease. Whether the dysregulation of immune system enhances oncogenesis or not remains obscure. Until now, no nationwide population-based study has been conducted regarding this. As such, this paper aims to clarify cancer risk in vitiligo patients. A retrospective nationwide population-based cohort study between 2000 and 2010 was performed based on data from the National Health Insurance Research Database of Taiwan. Standardized incidence ratios (SIRs) of cancers were analyzed. Among the 12,391 vitiligo patients (5364 males and 7027 females) and 48,531.09 person-years of observation, a total of 345 cancers were identified. Significantly increased SIRs were observed for prostate cancer in male patients, thyroid cancer and breast cancer in female patients and bladder cancers in both male and female patients. Unfortunately, the low incidence rate of certain cancers limited the power of our statistical analyses. This study demonstrated the patterns of malignancies in vitiligo patients of Taiwan. Compared with the general population, male patients had higher risks of prostate cancer and female patients had higher risks of thyroid cancer and breast cancer. The risks of bladder cancer were also increased in both male and female patients.

## 1. Introduction

Vitiligo affects people worldwide with a prevalence of 0.38%–1% among different populations [1,2]. It usually begins in childhood or young adulthood and the peak of onset is at 10–30 years. Vitiligo is an autoimmune disease characterized by destruction of melanocytes. An association of vitiligo with other autoimmune diseases, especially autoimmune thyroid diseases, is frequently found [3,4]. As the immune system affects oncogenesis greatly [5,6], it raises interest to elucidate how the dysregulated immune system in vitiligo patients influences cancer development. One hypothesis is that the hyperactivated immune system could suppress cancer cells. On the contrary, the chronic inflammation is also considered a predisposing factor for cancers. Previous studies showed multiple associations between autoimmune diseases and cancer [6]. However, no population-based cohort studies regarding cancer risk in vitiligo patients had been performed. Only a few studies have been conducted discussing the relationship between melanoma, non-melanoma skin cancer and vitiligo and the results were conflicting [7,8,9,10]. Therefore, we conducted a nationwide population-based cohort study to elucidate cancer risks in patients with vitiligo.

## 2. Materials and Methods

### 2.1. Data Source

In this study, we analyzed the published national data from the National Health Insurance Research Database (NHIRD) in Taiwan. Taiwan initiated a single-payer National Health Insurance (NHI) Program in 1995. Currently, there are more than 23 million enrollees in the program, representing approximately 99% of Taiwan’s entire population. The NHIRD, released and audited by the Department of Health and Bureau of the NHI program, provides comprehensive information about the insured subjects. The NHIRD has been extensively used in many epidemiologic studies in Taiwan [11,12,13,14]. The International Classification of Diseases, Ninth Revision, Clinical Modification (ICD-9-CM) was used by the board-certified physicians to define diseases. Personal information including family history, lifestyle and habits was not available from the NHIRD. 

### 2.2. Study Sample

This retrospective population-based cohort study made use of data between 2000 and 2010 from the NHIRD. Subjects with vitiligo diagnoses (ICD-9-CM code 709.01) were selected. Subjects were considered to have vitiligo only if the diagnosis was made by dermatologists and the condition required three or more outpatient visits. We excluded subjects with prior vitiligo diagnosis. Subjects with malignancy before the diagnosis of vitiligo were also excluded. A total of 12,391 vitiligo subjects were enrolled in the study group. Registrants with dubious basic data, such as conflicting gender, were not included. This study was approved by the research ethics board of Taipei Veterans General Hospital (VGHIRB No.: 2013-02-007AC).

### 2.3. Identification of Cancer Cases

We validated the diagnoses of cancers with the records from the Registry of Catastrophic Illness Patient Database, a separate subpart of the NHIRD. Insured patients who suffer from a major disease can apply for a catastrophic illness certificate, which grants exemption from all copayments. To apply for a cancer catastrophic illness certificate, cytological or pathological reports or evidence supporting the diagnosis is required. A catastrophic illness certificate is not issued to patients with in situ malignancies. The validity of using the Registry of Catastrophic Illness Patient Database has been assessed and previous studies have confirmed the high accuracy in detecting cancers [15]. The diagnostic codes of malignancies were defined as those from 140 to 208.91 in the ICD-9-CM format. Metastatic cancers in lymph nodes (ICD-9-CM codes 195-195.9) and secondary cancers (ICD-9-CM codes 196-199) were excluded from the study.

### 2.4. Cancer Risk Analysis

All enrolled patients were followed up from the date of a first-time diagnosis of vitiligo until a first-time diagnosis of cancer (except malignancy in situ, metastasis, or secondary cancer), death, the end of follow-up in the medical records, or 31 December 2010. The standardized incidence ratios (SIRs) of vitiligo-associated primary cancers were calculated. Stratified analyses were also conducted.

### 2.5. Sensitivity Analysis

Sensitivity analyses were conducted to evaluate the persistence of cancer risks in vitiligo patients, when we included all patients with at least two consecutive vitiligo diagnoses (ICD-9-CM code 709.01) made by dermatologists and excluded those with other diagnostic codes mimicking vitiligo, including tinea versicolor (ICD-9-CM code 111.0), secondary syphilis (ICD-9-CM codes 091.7, 091.3, 091.8, 091.9), albinism (ICD-9-CM code 270.2), lichen sclerosus et atrophicus and morphea (ICD-9-CM code 701.0).

### 2.6. Statistical Analysis

We examined the associations among vitiligo patients and specific cancer types using SIRs. SIR was calculated as the number of observed cancer cases among vitiligo patients divided by the expected number of cancer cases. The expected number of cancer cases was obtained from the product of national age-specific, gender-specific incidence rates obtained from the Taiwan National Cancer Registry [16] and the number of person-years at risk.

To assess the age effects on the relative risk of malignancies, we divided all enrollees into five age categories: <20 years, 20–39 years, 40–59 years, 60–79 years and >80 years at the date of a first-time diagnosis of vitiligo. We analyzed whether the association of post-vitiligo malignancies varied with the duration after first-time vitiligo diagnosis. Patients were classified into three groups based on duration of follow-up: <1 year, 1–5 years and 5–10 years. The 95% confidence interval (CI) of SIR was calculated utilizing Mid-P exact test. To minimize type I error, a two-tailed *p* value < 0.05 with Bonferroni correction was regarded as statistically significant.

Microsoft Office Excel 2003 and Microsoft SQL Server 2000 (Microsoft Corp., Redmond, WA, USA) were used to analyze the data in this study.

## 3. Results

### 3.1. Study Cohort

The total cohort consisted of 12,391 vitiligo patients (5364 men and 7027 women) with a mean age ± standard deviation (SD) at first visit of vitiligo of 42.8 ± 20.54 years (Table 1). The mean length ± SD of follow-up was 3.92 ± 2.10 years, with 48,531.09 person-years of follow-up. The most frequent length of follow-up was between 3 and 6 years, with 46.9% of patients.

### 3.2. Cancer Incidence

During the study period, a total of 345 cancers were identified. The overall incidence of malignancies was 0.71 per 100 person-years. The most common cancers in male vitiligo patients were prostate cancer, liver cancer and colorectal cancer, constituting 22.3%, 16.3% and 10.8% of the cancers, respectively (Table 2). As for female patients, the most common cancers were breast cancer, lung cancer and colorectal cancer, constituting 35.8%, 11.7% and 10.1% of the cancers, respectively.

### 3.3. SIRs of All Cancers in Patients with Vitiligo

The cancer risks in vitiligo patients were compared to those of the general population and the SIRs were calculated (Table 3). The overall cancer risks were elevated in vitiligo patients (SIR 1.75, 95% CI 1.574–1.944). For all vitiligo patients, the SIR of bladder cancer (SIR 3.65, 95% CI 2.348–5.441), thyroid cancer (SIR 3.19, 95% CI 1.917–4.995) and breast cancer (SIR 2.59, 95% CI 2.015–3.28) were increased. Although the SIRs of melanoma, cancers of oral cavity, hepatoma and lymphoma were also increased in vitiligo patients, the significance was lost after correction for multiple testing. No specific cancer occurred less frequently in vitiligo patients than in the general population.

When stratified by sex, male patients had a 3.2-fold increased risk of bladder cancer (SIR 3.21, 95% CI 1.872–5.259) and a 3.1-fold increased risk of prostate cancer (SIR 3.10, 95% CI 2.217–4.233). Female patients had a 4.8-fold increased risk of bladder cancer (SIR 4.82, 95% CI 2.237–9.148), a 3.4-fold increased risk of thyroid cancer (SIR 3.39, 95% CI 1.971–5.468) and a 2.6-fold increased risk of breast cancer (SIR 2.56, 95% CI 1.988–3.247). No specific cancer occurred less frequently in vitiligo patients than in the general population.

### 3.4. Stratified SIR Analysis by Age, Sex and Duration of Follow-Up

We analyzed SIRs of thyroid cancer, breast cancer, bladder cancer and prostate cancers, with stratification by age, sex and duration of follow-up (Table 4). For all patients, the highest risk of bladder cancer was in patients at the ages of 60–79 (SIR 3.43, 95% CI 1.907–5.716) and within one year of follow-up (SIR 50.71, 95% CI 16.11–122.3). For female patients, the highest risks were the ages of 20–39 for thyroid cancer (SIR 8.41, 95% CI 3.904–15.96) and <20 for breast cancer (SIR 672, 95% CI 33.63–3314). Risks of both thyroid cancer (SIR 35.24, 95% CI 54.908–116.4) and breast cancer (SIR 45.71, 95% CI 26.56–73.7) peaked within one year of follow-up. As for male patients, the risk of prostate cancer peaked in patients at the ages of >80 (SIR 4.16, 95% CI 2.254–7.072) and within one year of follow-up (SIR 69.69, 95% CI 35.35–124).

### 3.5. Sensitivity Analyses

When at least two diagnoses of vitiligo were required and other diagnostic codes masquerading as vitiligo were excluded, most elevated cancer risks in vitiligo patients persisted in comparison to the original results (Table 3), including the increased risk of prostate cancer in male patients (SIR 3.36, 95% CI 2.542–4.361), the increased risks of thyroid cancer (SIR 3.15, 95% CI 1.975–4.772) and breast cancer in female patients (SIR 2.53, 95% CI 2.047–3.089) and the increased risk of bladder cancer in male patients (SIR 3.27, 95% CI 2.024–5.005). However, the trend of increased risk of bladder cancer in female vitiligo patients became statistically insignificant after Bonferroni correction (SIR 3.27, 95% CI 1.518–6.208).

## 4. Discussion

In the present study, 12,391 vitiligo patients with female predominance were enrolled. The mean age at first visit ± standard deviation was 42.8 ± 20.54 years. Previous studies in vitiligo patients indicated a peak of onset at 10–30 years of age [2,17]. Therefore, our studies suggested that there might be a delayed visit of medical resources after the onset of the disease, probably due to patients’ indifference toward this asymptomatic disease and less severity of disease at onset. A female preponderance may reflect greater cosmetic concerns in women.

The overall incidence of post-vitiligo malignancies was 0.71 per 100 person-years, slightly elevated when compared to that in the general population (0.28 per 100 person-years) and the SIRs of post-vitiligo malignancies showed significant elevation, without gender difference. Elevated cancer risk of bladder cancer was demonstrated for all vitiligo patients. Regarding the impact of sex, male vitiligo patients were at increased risk of prostate cancer. Conversely, female vitiligo patients had higher risks of thyroid cancer and breast cancer.

Although the relationship between vitiligo and malignancies was not well-established, various cases of malignant tumors have been reported in association with vitiligo, including melanoma [18,19], squamous cell carcinoma [20], basal cell carcinoma [21], breast cancer [22,23], bladder cancer [24], colorectal cancer [25], leukemia [26] and Hodgkin’s disease [27]. Our nationwide population-based study provided evidence on the increased risks of certain cancer in vitiligo patients. Although the underlying mechanisms remain elusive, some hypotheses might partially explain our findings, including dysregulated immune system and chronic inflammation, hormonal carcinogenesis and vitamin D deficiency.

Prior research has shown global activation of melanocyte-specific CD8+ cytotoxic T-lymphocytes (CTLs) in vitiligo-induced depigmentation [28]. In addition, CD8+ CTLs also play a crucial role in tumor immunity and aberrant CD8+ CTLs infiltration-induced focal tumor capsule disruption is associated with cancer invasion and metastasis [29,30]. The aberrant activated CD8+ CTLs may provide a link between cancer and vitiligo.

Several case studies have suggested the association between urinary bladder cancer and vitiligo. One report showed that squamous cell carcinoma of the bladder may present as vitiligo [24] and another disclosed an increased risk of urological cancer after all autoimmune diseases [31]. However, there has been limited published evidence supporting the association between vitiligo and bladder cancer. In our study, an increased risk of thyroid cancer was revealed in female patients with vitiligo. The possible association between vitiligo, autoimmune thyroid disease and thyroid cancer had also been postulated in previous studies [32,33]. The dysregulated CD8+ CTLs and vitiligo-associated autoimmune thyroid diseases may play a role in the thyroid cancer risks in vitiligo patients. Regarding breast cancer, increased risks were observed in female vitiligo patients. Vitiligo has been found to be associated with breast cancer, although studies remain limited to some case reports [19,20,34]. Function-blocking autoantibodies to the melanin-concentrating hormone receptor have been identified in vitiligo patients [35]. Successful treatment of vitiligo with a sex steroid-thyroid hormone mixture was also demonstrated [36]. Hence, the potential role of hormones in the carcinogenesis might partially explain the increased risks of breast cancer in vitiligo patients. Due to the limited knowledge on the association between vitiligo and breast cancer, further studies are needed to confirm our findings. In our study, male vitiligo patients had a 3.1-fold increased risk of prostate cancer. Although the association between prostate cancer and vitiligo has not been reported previously, some studies have suggested the roles of ultraviolet irradiance and vitamin D in the risk of prostate cancer. Several studies indicated that vitamin D metabolites have an antiproliferative and a pro-differentiating effect on prostate cancer cell lines and that vitamin D deficiency is associated with prostate cancer risk [37,38]. Meanwhile, vitamin D deficiency has been linked to the development of autoimmune disorders [39,40]. In one study, 55.6% of vitiligo patients had insufficient 25-hydroxyvitamin D level in the sera [41] and topical vitamin D analogues have been used as an alternative treatment of vitiligo [42]. Therefore, deficiency of vitamin D might be associated with the increased risks of prostate cancer in vitiligo patients, while more studies are needed to investigate the association between these two diseases.

Considering that patients with vitiligo have decreased melanocytes and less protective pigment in vitiligo lesions, it would be expected that these patients have higher risks of skin cancer. The risk for skin cancer in vitiligo is still being debated [7]. Studies in Caucasian populations showed conflicting results [8,9,10]. In our study, we observed a decreased risk of NMSC and an increased risk of melanoma in vitiligo patients, while no statistical significance was found. Further studies are necessary to clarify the mechanisms underlying the ethno-racial differences.

In analysis of the SIRs of cancer with stratification by age and duration of follow-up, the SIRs peaked ubiquitously within one year of follow-up. Further analysis showed that the risks of prostate cancer in males, breast cancer and thyroid cancer in females and bladder cancer in both males and females remained increased until 5 years of follow-up. This finding might suggest a potential link between vitiligo and the aforementioned cancers. However, we should be cautious to evaluate the temporal relationship between vitiligo and these cancers, because aggressive surveillance for cancer during that period of time may have resulted in detection bias.

Our study has several limitations. First, the NHIRD did not contain some personal information, such as family history of malignancies, smoking, alcohol use, etc., which may serve as risk factors of certain cancers. Second, misclassification of disease cannot be ruled out in a registry-based data set. To diminish the possible misdiagnosis of vitiligo cases, we enrolled only those with at least three consecutive vitiligo diagnoses made by dermatologists. Third, the low prevalence of vitiligo in Taiwan and the low incidence rate of certain cancers led to a small number of enrolled cases, thus limiting the statistical power in our study.

## 5. Conclusions

In summary, this nationwide population-based study demonstrated that compared with the general population, male patients had higher risks of prostate cancer and female patients had higher risks of thyroid cancer and breast cancer. The risks of bladder cancer were also increased in both male and female patients. At present, it is not known whether the coexistence of vitiligo and various malignant tumors is merely a coincidence or whether there are shared etiologic factors. Further studies are needed to address this issue.

## Figures and Tables

**Table 1:** Characteristics of vitiligo patients.

Patient Characteristics	N	(%)
Total patient no.	12,391	
Gender		
Male	5364	(43.3)
Female	7027	(56.7)
Age of first visit, mean ± SD	42.8 ± 20.54	
<20 years	2034	(16.4)
20–29 years	1673	(13.5)
30–39 years	1780	(14.4)
40–49 years	2070	(16.7)
50–59 years	2121	(17.1)
60–69 years	1357	(11.0)
70–79 years	1058	(8.5)
>80 years	298	(2.4)
Total person-years	48,531.09	
Male	20,995.68	
Female	27,535.41	
Duration of follow-up *, years		
<1	1193	(9.6)
1–3	3116	(25.1)
3–6	5811	(46.9)
>6	2271	(18.3)

Abbreviations: SD, standard deviation. * Duration of follow-up was calculated from the date of first diagnosis of vitiligo to the date of a first-time diagnosis of cancer, death, the end of follow-up in the medical records, or the end of 2010.

**Table 2:** Types of post-vitiligo malignancies.

Cancer Site	Total, N	(%)	Male, N	(%)	Female, N	(%)
Oral cavity *	22	(6.4)	17	(10.2)	5	(2.8)
NPC	6	(1.7)	5	(3.0)	1	(0.6)
Digestive						
Stomach	17	(4.9)	12	(7.2)	5	(2.8)
Liver	43	(12.5)	27	(16.3)	16	(8.9)
Colorectum ^†^	36	(10.4)	18	(10.8)	18	(10.1)
Pancreas	1	(0.3)	0		1	(0.6)
Urinary tract						
Kidney	4	(1.2)	2	(1.2)	2	(1.1)
Bladder cancer	22	(6.4)	14	(8.4)	8	(4.5)
Respiratory						
Lung ^‡^	35	(10.1)	14	(8.4)	21	(11.7)
Skin						
NMSC	2	(0.6)	0		2	(1.1)
Melanoma	3	(0.9)	2	(1.2)	1	(0.6)
Endocrine						
Thyroid	17	(4.9)	2	(1.2)	15	(8.4)
Hematological						
Lymphoma	14	(4.1)	9	(5.4)	5	(2.8)
Leukemia	7	(2.0)	6	(3.6)	1	(0.6)
Male cancers						
Prostate	37	(10.7)	37	(22.3)	N/A	
Female cancers						
Uterus	14	(4.1)	N/A		14	(7.8)
Breast	65	(18.8)	1	(0.6)	64	(35.8)
All cancer	345	(100)	166	(100)	179	(100)

Abbreviations: NMSC, non-melanoma skin cancer; NPC, nasopharyngeal carcinoma; N/A: non-applicable. * Oral cavity, including lip, tongue, gum, floor of mouth, oropharynx, hypopharynx and salivary glands. ^†^ Colorectum, including colon, rectum, rectosigmoid junction and anus. ^‡^ Lung, including trachea, bronchus and lung.

**Table 3:** Standardized incidence ratios for all cancers in 12,391 vitiligo patients.

Site of Cancers	Total			Male			Female		
	O/E	SIR	(95% CI)	O/E	SIR	(95% CI)	O/E	SIR	(95% CI)
Oral cavity *	22/12.49	1.76	(1.132–2.624)	17/11.13	1.53	(0.919–2.395)	5/1.35	3.7	(1.354–8.191)
NPC	6/3.49	1.72	(0.698–3.581)	5/2.33	2.15	(0.786–4.757)	1/1.16	0.87	(0.043–4.268)
Digestive									
Stomach	17/10.57	1.61	(0.968–2.523)	12/6.75	1.78	(0.963–3.021)	5/3.82	1.31	(0.480–2.905)
Liver	43/29.20	1.47	(1.079–1.965)	27/19.07	1.42	(0.952–2.031)	16/10.13	1.58	(0.935–2.511)
Colorectal ^†^	36/32.19	1.12	(0.795–1.532)	18/17.87	1.01	(0.616–1.561)	18/14.31	1.26	(0.769–1.949)
Pancreas	1/4.46	0.22	(0.011–1.107)	0/2.53	-		1/1.93	0.52	(0.026–2.559)
Urinary									
Kidney	4/2.44	1.64	(0.521–3.952)	2/1.55	1.29	(0.216–4.257)	2/0.89	2.25	(0.377–7.43)
Bladder	**22/6.02**	**3.65**	**(2.348–5.441)**	**14/4.36**	**3.21**	**(1.827–5.259)**	**8/1.66**	**4.82**	**(2.237–9.148)**
Respiratory									
Lung ^‡^	35/29.09	1.2	(0.851–1.655)	14/18.72	0.75	(0.426–1.225)	21/10.37	2.03	(1.287–3.043)
Skin									
NMSC	2/4.33	0.46	(0.077–1.526)	0/2.38	-		2/1.95	1.02	(0.172–3.383)
Melanoma	3/0.55	5.41	(1.376–14.72)	2/0.29	6.97	(1.168–23.02)	1/0.27	3.74	(0.187–18.42)
Endocrine									
Thyroid	**17/5.34**	**3.19**	**(1.917–4.995)**	2/0.92	2.19	(0.366–7.221)	**15/4.42**	**3.39**	**(1.971–5.468)**
Hematological									
Lymphoma	14/5.64	2.48	(1.412–4.064)	9/3.06	2.94	(1.432–5.39)	5/2.58	1.94	(0.711–4.298)
Leukemia	7/4.33	1.62	(0.708–3.2)	6/2.38	2.53	(1.024–5.254)	1/1.95	0.51	(0.026–2.528)
Male cancers									
Prostate	N/A	N/A		**37/11.92**	**3.1**	**(2.217–4.233)**	N/A	N/A	
Female cancers									
Uterus	N/A	N/A		N/A	N/A		14/9.79	1.43	(0.815–2.346)
Breast	**65/25.10**	**2.59**	**(2.015–3.28)**	1/0.09	10.74	(0.538–52.99)	**64/25.01**	**2.56**	**(1.988–3.247)**
All cancers	**345/196.92**	**1.75**	**(1.574–1.944)**	**166/105.36**	**1.58**	**(1.349–1.829)**	**179/91.57**	**1.96**	**(1.684–2.257)**

Abbreviations: CI, confidence interval; E, expected number of cancer cases, based on estimates of general population in Taiwan in 2008, adjusted for age and gender; NPC, nasopharyngeal carcinoma; N/A, not applicable; NMSC, non-melanoma skin cancer; O, observed number of cancer cases; SIR, standardized incidence ratio. * Oral cavity, including lip, tongue, gum, floor of mouth, oropharynx, hypopharynx and salivary glands. ^†^ Rectum, including rectum, rectosigmoid junction and anus. ^‡^ Lung, including trachea, bronchus and lung. Numbers in bold type indicate statistical significance.

**Table 4:** Standardized incidence ratios for selected cancers in vitiligo patients, stratified by age at the date of a first-time diagnosis and duration of follow-up.

Characteristics	Bladder Cancer			Breast Cancer			Thyroid Cancer			Prostate Cancer		
	Total Patients			Female Patients			Female Patients			Male Patients		
	O/E	SIR	(95% CI)	O/E	SIR	(95% CI)	O/E	SIR	(95% CI)	O/E	SIR	(95% CI)
Age at first visit, years												
<20	0/0.003	-		**1/0.0012**	**672**	**(33.63–3314)**	0/0.04	-		0/0	-	
20–39	2/0.08	26.4	(4.425–87.21)	6/1.93	3.12	(1.264–6.484)	**8/0.95**	**8.41**	**(3.904–15.96)**	0/0.01	-	
40–59	5/0.92	5.41	(1.982–11.99)	**32/15.71**	**2.04**	**(1.417–2.842)**	4/2.33	1.72	(0.545–4.137)	3/0.48	6.3	(1.604–17.16)
60–79	**13/3.79**	**3.43**	**(1.907–5.716)**	**25/7.07**	**3.53**	**(2.338–5.14)**	3/1.06	2.84	(0.723–7.732)	**22/8.55**	**2.57**	**(1.653–3.83)**
>80	2/1.22	1.64	(0.275–5.424)	0/0.30	-		0/0.03	-		**12/2.88**	**4.16**	**(2.254–7.072)**
Duration of follow-up * years												
<1	**4/0.08**	**50.71**	**(16.11–122.3)**	**15/0.33**	**45.71**	**(26.56–73.7)**	**2/0.06**	**35.24**	**(5.908–116.4)**	**10/0.14**	**69.59**	**(35.35–124)**
1–5	**17/2.83**	**6.01**	**(3.618–9.427)**	**44/11.90**	**3.7**	**(2.719–4.917)**	**11/2.08**	**5.3**	**(2.787–9.212)**	**23/5.45**	**4.22**	**(2.742–6.237)**
5–10	1/3.50	0.29	(0.014–1.409)	5/14.21	0.35	(0.129–0.780)	2/2.89	0.69	(0.116–2.283)	4/6.89	0.58	(0.184–1.4)
Average of follow-up duration, days												
	887.45			1002.52			1193.33			901.27		

Abbreviations: CI, confidence interval; E, expected number of cancer cases, based on estimates of general population in Taiwan in 2008, adjusted for age and gender; O, observed number of cancer cases; SIR, standardized incidence ratio. * Duration of follow-up was calculated from the date of a first-time diagnosis of vitiligo until a first-time diagnosis of cancer, death, the end of follow-up in the medical records, or the end of 2010. Numbers in bold type indicate statistical significance.
